# Dynamic accumulation of fatty acids in duck (*Anas platyrhynchos*) breast muscle and its correlations with gene expression

**DOI:** 10.1186/s12864-020-6482-7

**Published:** 2020-01-17

**Authors:** Wenlei Fan, Wenjing Liu, Hehe Liu, Qingshi Meng, Yaxi Xu, Yuming Guo, Baowei Wang, Zhengkui Zhou, Shuisheng Hou

**Affiliations:** 1 Key Laboratory of Animal (Poultry) Genetics Breeding and Reproduction, Ministry of Agriculture and Rural Affairs; State Key Laboratory of Animal Nutrition, Institute of Animal Science, Chinese Academy of Agricultural Sciences, No. 2 Yuanmingyuan W Rd, Beijing, 100193 China; 2 College of Food Science and Engineering, Qingdao Agricultural University, Qingdao, 266109 People’s Republic of China; 3 College of Animal Science and Technology, China Agricultural University, Beijing, 100193 People’s Republic of China

**Keywords:** Lipid metabolism, Fatty acid profile, Duck, Breast muscle, Transcriptome

## Abstract

**Background:**

Fatty acid composition contributes greatly to the quality and nutritional value of meat. However, the molecular regulatory mechanisms underlying fatty acid accumulation in poultry have not yet been cleared. The aims of this study were to characterize the dynamics of fatty acid accumulation in duck breast muscle and investigate its correlations with gene expression.

**Results:**

Here, we analyzed the fatty acid profile and transcriptome of breast muscle derived from Pekin ducks and mallards at the ages of 2 weeks, 4 weeks, 6 weeks and 8 weeks. Twenty fatty acids were detected in duck breast muscle, with palmitic acid (C16:0, 16.6%~ 21.1%), stearic acid (C18:0, 9.8%~ 17.7%), oleic acid (C18:1n-9, 15.7%~ 33.8%), linoleic acid (C18:2n-6, 10.8%~ 18.9%) and arachidonic acid (C20:4n-6, 11.7%~ 28.9%) as the major fatty acids. Our results showed that fatty acid composition was similar between the two breeds before 6 weeks, but the compositions diverged greatly after this point, mainly due to the stronger capacity for C16:0 and C18:1n-9 deposition in Pekin ducks. By comparing the multistage transcriptomes of Pekin ducks and mallards, we identified 2025 differentially expressed genes (DEGs). Cluster analysis of these DEGs revealed that the genes involved in oxidative phosphorylation, fatty acid degradation and the PPAR signaling pathway were upregulated in mallard at 8 weeks. Moreover, correlation analysis of the DEGs and fatty acid composition traits suggested that the DEGs involved in lipogenesis, lipolysis and fatty acid *β*-oxidation may interact to influence the deposition of fatty acids in duck breast muscle.

**Conclusions:**

We reported the temporal progression of fatty acid accumulation and the dynamics of the transcriptome in breast muscle of Pekin ducks and mallards. Our results provide insights into the transcriptome regulation of fatty acid accumulation in duck breast muscle, and will facilitate improvements of fatty acid composition in duck breeding.

## Background

Poultry meat is among the most common animal sources of food, accounting for approximately 30% of meat consumption worldwide. In recent decades, meat quality has become an increasingly important factor influencing consumer preferences. Intramuscular fat (IMF) content and its fatty acid composition are important factors determining meat quality, by affecting flavor, juiciness, tenderness, muscle color and overall liking [1–3]. Diets rich in monounsaturated fatty acids (MUFAs) and polyunsaturated fatty acids (PUFAs) can decrease the risks of cardiovascular disease and diabetes in humans [4, 5]. Additionally, PUFAs have a marked tendency to be oxidized, producing a rancid odor and taste that decrease consumer acceptance [6]. Therefore, ways to manipulate the fatty acid composition of meat are valuable.

It has been widely reported that the fatty acid composition of meat can be affected by various factors such as age, sex, and rearing conditions of the animals [7–10]. In addition, fatty acid compositions are heritable traits, with heritability ranging between 0.2 and 0.6 in various populations of pigs [11, 12]. Chickens and ducks of different breeds have been shown to vary in fatty acid composition, suggesting that genetic factors may influence fatty acid composition, and breeding poultry for favorable fatty acid composition is possible [13, 14].

Duck (*Anas platyrhynchos*) is one of the economically important domestic fowls providing meat, eggs and feathers to humans. Compared with the phenotypes of their wild ancestors (mallards), the phenotypes of Pekin ducks have diverged significantly due to intensive artificial selection. The divergent phenotypes of Pekin ducks include white plumage, extraordinary body size, large deposits of sebum, excellent muscle yield performance and high IMF content. Consequently, in addition to having economic value, the Pekin duck provides a powerful system for dissecting artificial selection mechanisms in farm animals. In our previous study, we identified the mechanisms leading to white plumage and enlarged body size in Pekin ducks using this system [15]. It has been reported that the IMF content in Pekin duck was approximately 20% higher than that in mallard [16]. However, the fatty acid composition of IMF in ducks and the underlying molecular mechanisms remain poorly understood.

The accumulation of fatty acids in muscle is a dynamic process that is regulated by multiple biological processes, including lipogenesis, fatty acid uptake and fatty acid *β*-oxidation [17–20]. Large efforts have been made to identify the genes and gene networks associated with fatty acid composition traits in pigs and cattle [21–23]. In addition, several works have aimed to understand the lipid deposition in breast muscle of poultry using approaches such as transcriptomic, proteomic and metabolomic analysis. Transcriptome analysis of chicken breast muscle over a time course revealed the relationships of IMF deposition with various pathways, such as *β*-oxidation of fatty acids and PPAR signaling pathways [24, 25]. However, on their own, transcriptome or other omics data have limitations for predicting lipid metabolism. The integration of transcriptomic data and fatty acid profiles over a time course can increase our understanding of lipid accumulation in the breast muscle of poultry.

To explore the genes and pathways associated with fatty acid composition in ducks, we analyzed the fatty acid profile and transcriptome of breast muscle of Pekin duck and mallard at the ages of 2 weeks, 4 weeks, 6 weeks and 8 weeks. The investigation of gene expression patterns and their correlations with fatty acid composition traits suggested that the increased IMF content in Pekin duck is the result of multiple metabolic processes rather than the consequence of a single biochemical event. Together, our results provide important insights into the potential mechanisms that affect lipid metabolism and IMF content in duck breast muscle, especially from a temporal perspective.

## Results

### Compositions of fatty acids in breast muscle of Pekin duck and mallard

**Fig. 1:**
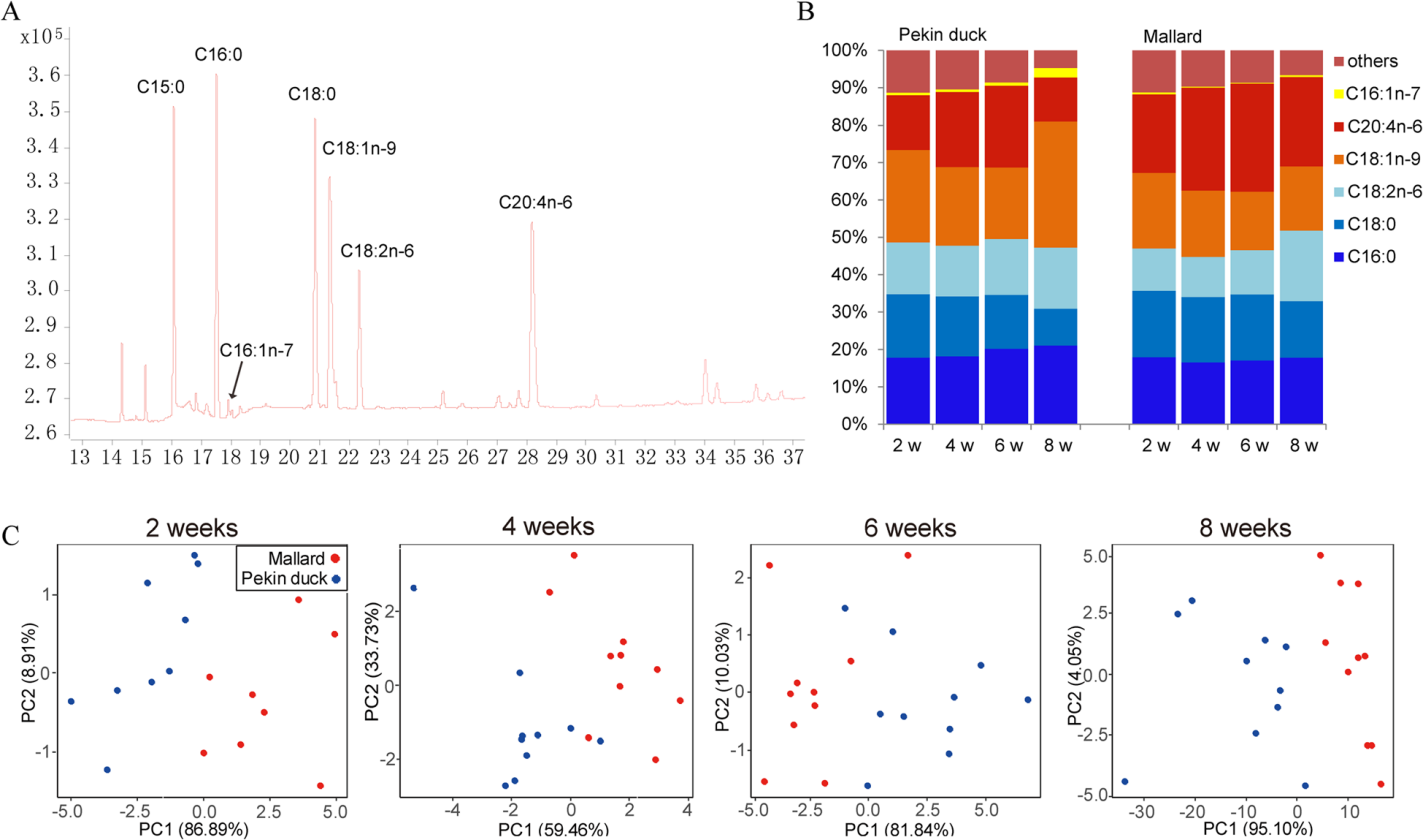
Composition of fatty acids in breast muscle of Pekin ducks and mallards (**a**) Representative GC chromatograms of fatty acids in duck breast muscle (only the major fatty acids are marked). **b** Percentage of major fatty acid species at different developmental stages. **c** PCA analysis of fatty acid content at different development stages

We assessed the temporal progression of lipid accumulation in the breast muscle of Pekin ducks and mallards by measuring the fatty acid profiles at four developmental time points ranging from 2 weeks to 8 weeks post-hatch (2 weeks, 4 weeks, 6 weeks, 8 weeks). Gas chromatography analysis were performed to characterize the fatty acid profiles of breast muscle, and 20 fatty acids were detected (Fig. 1a, Additional file 1). The palmitic acid (C16:0, 16.6%~ 21.1%), stearic acid (C18:0, 9.8%~ 17.7%), oleic acid (C18:1n-9, 15.7%~ 33.8%), linoleic acid (C18:2n-6, 10.8%~ 18.9%) and arachidonic acid (C20:4n-6, 11.7%~ 28.9%) were the major fatty acids in duck breast muscle, together accounting for more than 88% of the total fatty acid content (TFA, sum of all identified fatty acids).

Unlike the mallards, the Pekin ducks had high percentages of palmitic and oleic acid but low percentages of arachidonic acid, especially at 8 weeks (Fig. 1b). The fatty acid compositions of the two breeds were relatively similar to each other before 6 weeks, but differed greatly at 8 weeks. Principal component analysis (PCA) of fatty acid concentration revealed that the two breeds could be clearly separated into different clusters at 2 weeks and 8 weeks, but not at 4 weeks or 6 weeks (Fig. 1c). These results suggest that both genetics and developmental stages may influence the fatty acid composition of duck breast muscle.

### Effects of sex on fatty acid composition of duck breast muscle

To characterize the difference in the fatty acid profiles of IMF between male and female ducks, we compared the relative content and percentage of each fatty acid using T-test (Additional file 2). For the relative content, the duck sex has no influence on the major fatty acid and fatty acid groups in both Pekin duck and mallard at almost all time point (*P* > 0.05). We observed that the relative content of SFA and TFA were higher in male than female mallard at 2 weeks (*P* < 0.05). In contrast, the relative content of C16:0, C18:0, C18:1n-9 and C18:2n-6, SFA, MUFA, PUFA and TFA were higher in male Pekin ducks than in females at 6 weeks (*P* < 0.05). The duck sex showed no influence on the composition of major fatty acids and fatty acid groups in both Pekin duck and mallard (*P* > 0.05), except that the male Pekin ducks showed a lower percentage of C20:4n-6 than females at 8 weeks (*P* < 0.05).

### Dynamic accumulation of fatty acids in breast muscle of Pekin duck and mallard

**Fig. 2:**
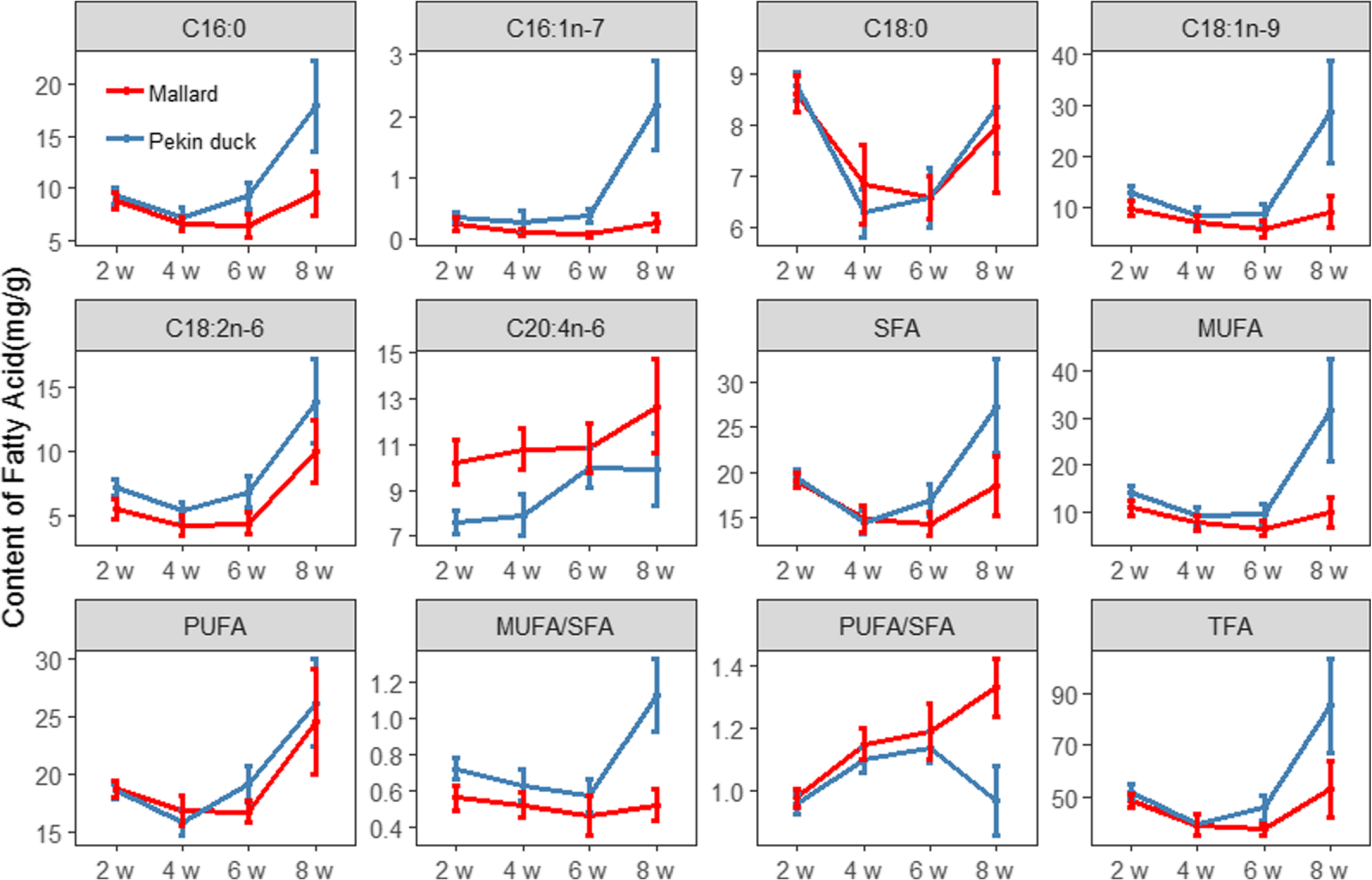
Dynamics of major fatty acids and fatty acid groups in breast muscle of Pekin ducks and mallards (means ± SD, *n* = 9 or 10)**.** SFA, MUFA and PUFA represent the sum of saturated, monounsaturated and polyunsaturated fatty acids, respectively. TFA represents the sum of all detected fatty acids. MUFA/SFA and PUFA/SFA represents the ratio of summed MUFA and PUFA with SFA, respectively (values has no unit)

**Fig. 3:**
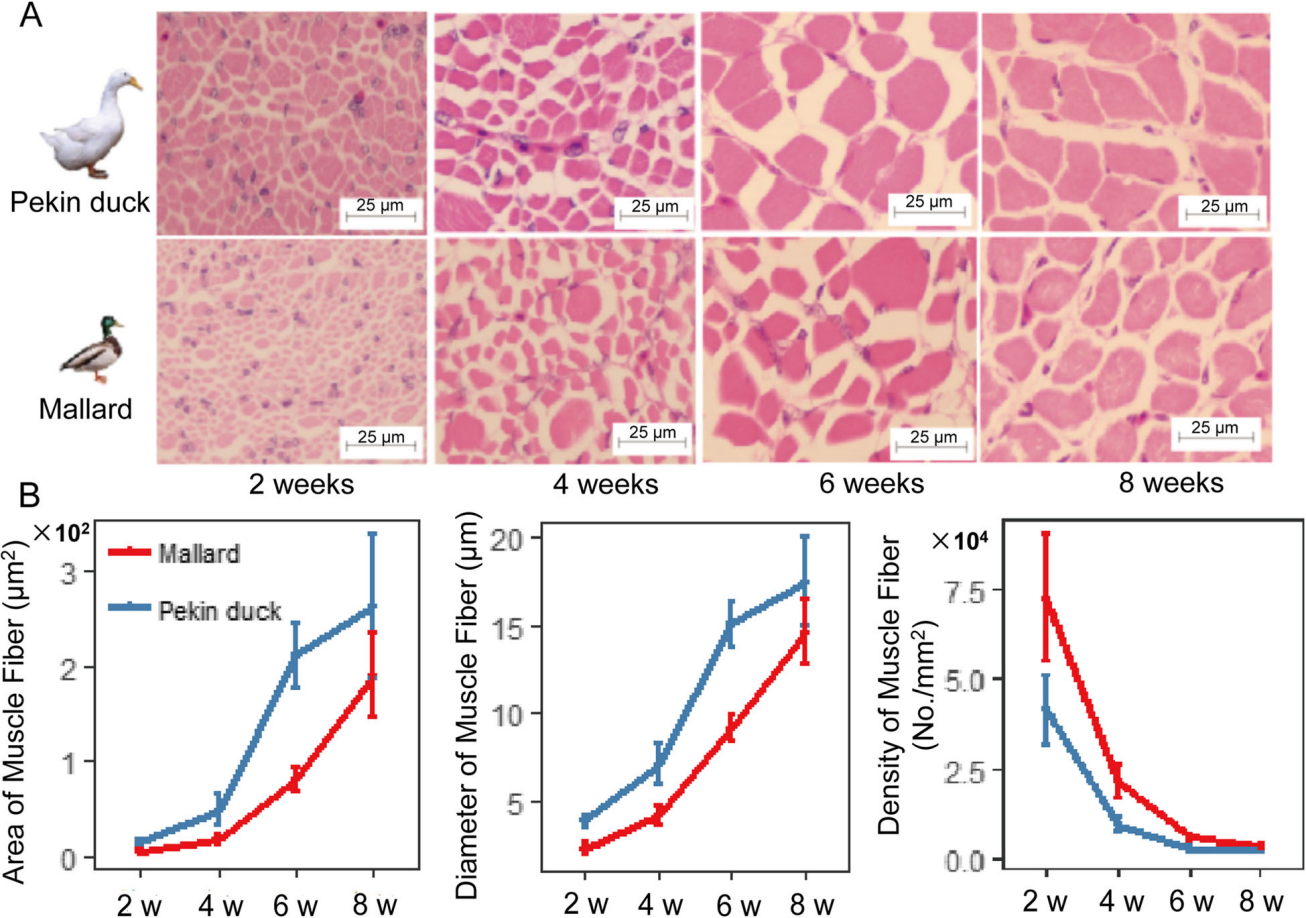
Histological analysis of breast muscle. **a** H&E staining of breast muscle at different developmental stages (**b**) Size (area, diameter) and density of muscle fibers over the course of development (means ± SD, n = 9 or 10;)

The contents of TFA, the majority of fatty acid groups and individual fatty acids decreased from 2 weeks to 4 weeks, remained largely steady from 4 weeks to 6 weeks, and then increased rapidly after 6 weeks in both breeds. However, from 2 weeks to 8 weeks, the content of C20:4 n-6 increased continuously, and the contents of several low-content fatty acids continuously decreased (Fig. 2, Additional file 3). From 6 weeks to 8 weeks, the accumulation speed of SFAs (mainly C16:0) and MUFAs (mainly C16:1n-7 and C18:1n-9) in Pekin duck exceeds that of mallard, whereas the mallards tended to accumulate PUFAs, especially C20:4n-6 (Fig. 2). Moreover, the speed of fatty acid accumulation is exactly the opposite of muscle fiber hypertrophy. Here, we observed that the increases in muscular histological traits such as the diameter and area of muscle fibers were greatest between 4 weeks and 6 weeks, and slowed down after 6 weeks (Fig. 3).

The content of TFA in Pekin duck were similar to that in mallard before 6 weeks, but diverged markedly thereafter. The difference in TFA content between the two breeds peaked at 8 weeks, with the differences in the C16:0, C16:1n-7 and C18:1n-9 contents representing more than 95% of this difference. These fatty acids are mainly the products of de novo fatty acid biosynthesis and ∆^9^-desaturase. The contents of C16:0, C16:1n-7, and C18:1n-9 in Pekin ducks at 8 weeks were approximately 2, 9 and 3 times those in mallards, respectively (*P* < 0.01; Additional file 2).

### Transcriptome analysis and identification of DEGs

To identify the potential genes involved in the regulation of lipid deposition in duck breast muscle, time-course mRNA-seq was performed with three biological replicates for each breed at 2 weeks, 4 weeks, 6 weeks and 8 weeks after birth. The filtered reads were mapped to the duck reference genome. The numbers of genes expressed in Pekin ducks and mallards were 11,898 and 11,678, respectively. To validate RNA-seq results, six genes of different expression level: acyl-CoA synthetase bubblegum family member 2 (*ACSBG2*), fatty acid synthase (*FASN*), acyl-CoA dehydrogenase long chain (*ACADL*), stearoyl-CoA desaturase (*SCD*), fatty acid binding protein 3 (*FABP3*) and lipoprotein lipase (*LPL*) were selected randomly and Q-PCR were performed to analyze the expression level of each gene at 6-weeks and 8-weeks for both breeds. The fold changes of the above six genes in RNA-seq and Q-PCR were related using Spearman rank correlation. A good concordance were observed between Q-PCR and RNA-seq (R^2^ = 0.87), which indicate that the RNA-seq results were reliable and appropriate for further analysis (Additional file 4).

**Fig. 4:**
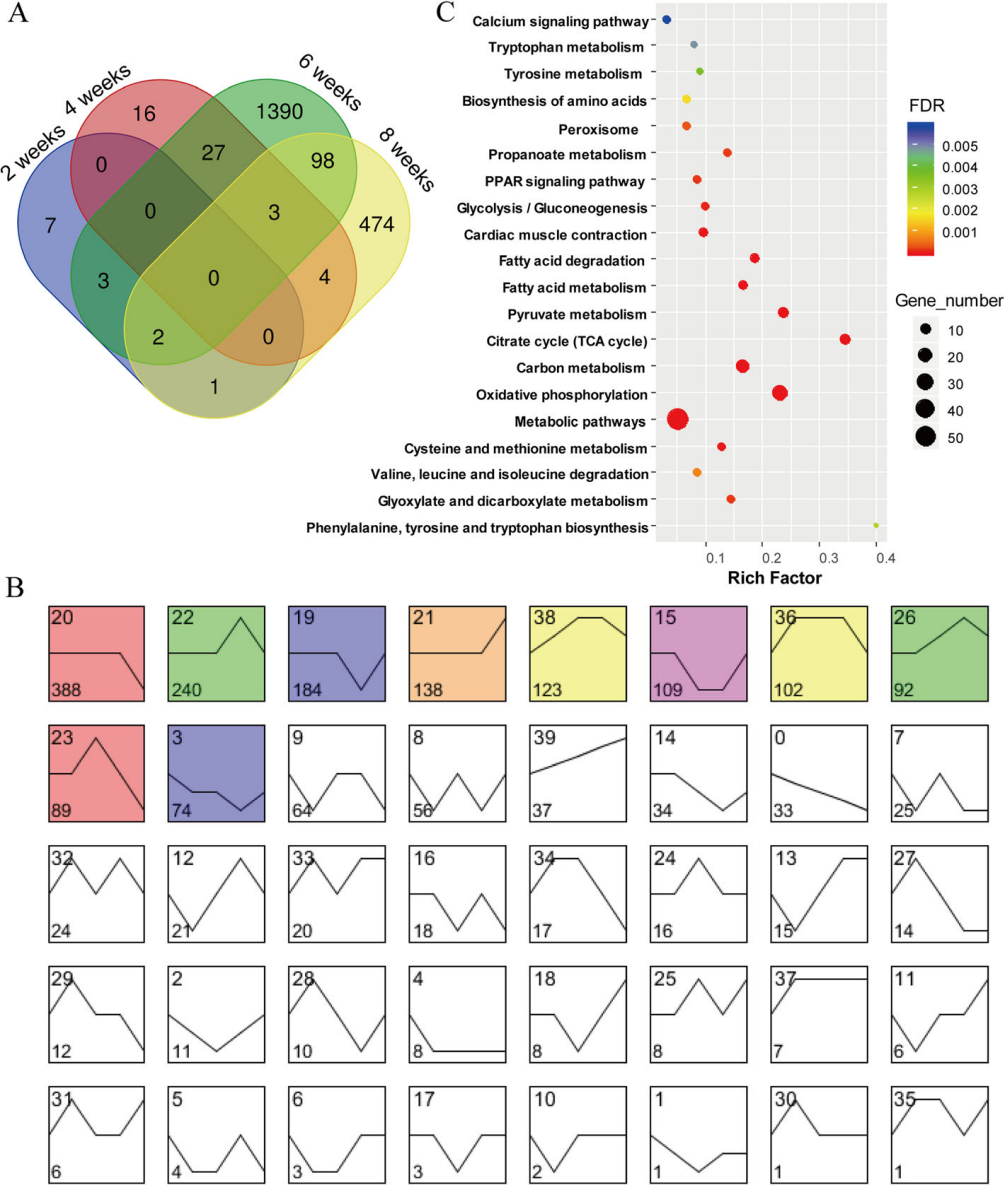
Identification and functional annotation of DEGs (**a**) Venn diagram of unique and shared DEG numbers in the same time point. **b** Short time-series expression miner (STEM) clustering of DEGs. All profiles are ordered based on the number of genes assigned (number at the bottom of each profile) and the significant profiles are colored. **c** KEGG pathway analysis of DEGs in profile21

Comparison of the two breed obtained 2024 differentially expressed genes (DEGs), and the numbers of DEGs at 2 weeks, 4 weeks, 6 weeks and 8 weeks were 13, 50, 1523 and 582, respectively. The number of DEGs markedly increased from 2 weeks to 6 weeks and decreased thereafter, suggesting large transcriptome changes before and after 6 weeks. This result is consistent with the dynamics of lipid accumulation and muscle fiber hypertrophy. We observed no DEGs that were common to two or more time points (Fig. 4a), indicating that the transcriptional regulation of breast muscle development and lipid deposition in muscle was temporally specific.

### Cluster analysis and functional annotation of DEGs

The 2024 DEGs were classified using Short Time-series Expression Miner software (STEM) based on their temporal expression patterns and a total of 10 significant profiles were obtained (Fig. 4b, Additional file 5). To examine whether a given expression pattern was linked to specific biological functions, enrichment analysis was performed to identify significantly overrepresented KEGG pathways among the genes in each profile. Of the 10 significant profiles, only profile 21 was observed to be closely linked to lipid metabolism. The representing KEGG pathway for this profile included oxidative phosphorylation (*P*_adjust_ = 4.02 × 10^− 33^, 27 genes), citrate cycle (*P*_adjust_ = 1.18 × 10^− 13^, 10 genes), fatty acid degradation (*P*_adjust_ = 3.27 × 10^− 07^, 6 genes) and the PPAR signaling pathway (*P*_adjust_ = 1.15 × 10^− 04^, 5 genes) (Fig. 4c, Additional file 5). The expression difference of genes in profile 21 remained largely steady before 6 weeks and then sharply increased from 6 weeks to 8 weeks, which implies that lipolysis of lipid in mallards may be higher than that in Pekin ducks during this stage.

The PPAR signaling pathway was also enriched in profile 19. Furthermore, the signaling pathway ECM-receptor interaction were enriched in profile 20 and profile 23, which has been identified as a candidate pathway that might participate in IMF accumulation during chicken development (Additional file 5). Despite several well-known lipogenesis related genes were included in different profiles, pathways related to fatty acid synthesis such as de novo fatty acid synthesis, fatty acid elongation and fatty acid desaturase were absent from the enrichment analysis of the 10 significant profiles. This absence may reflect the facts that gene expression patterns are extremely diverse and DEGs in one signaling pathway or with the same functions may occur in multiple profiles.

### Integration of transcriptome data and fatty acid profiles

To identify the associations between gene expression and traits, correlation analysis was performed on the abundances of transcripts and fatty acids or fatty acid groups. A total of nine fatty acid composition traits (C16:0, C18:0, C18:1n-9, C18:2n-6, C20:4n-6, SFA, MUFA, PUFA and TFA) and 2024 DEGs were subjected to Pearson correlation analysis, which revealed 18,216 gene–trait correlations (Additional file 6). After filtering, 513 genes were found to have strong correlation with at least one trait (|R| ≥ 0.7). Previous study has stated that causal relationships can not be inferred from gene–trait correlation analyses of fatty acid composition traits, because expression difference could be either cause or response of changes in the traits [26].

**Fig. 5:**
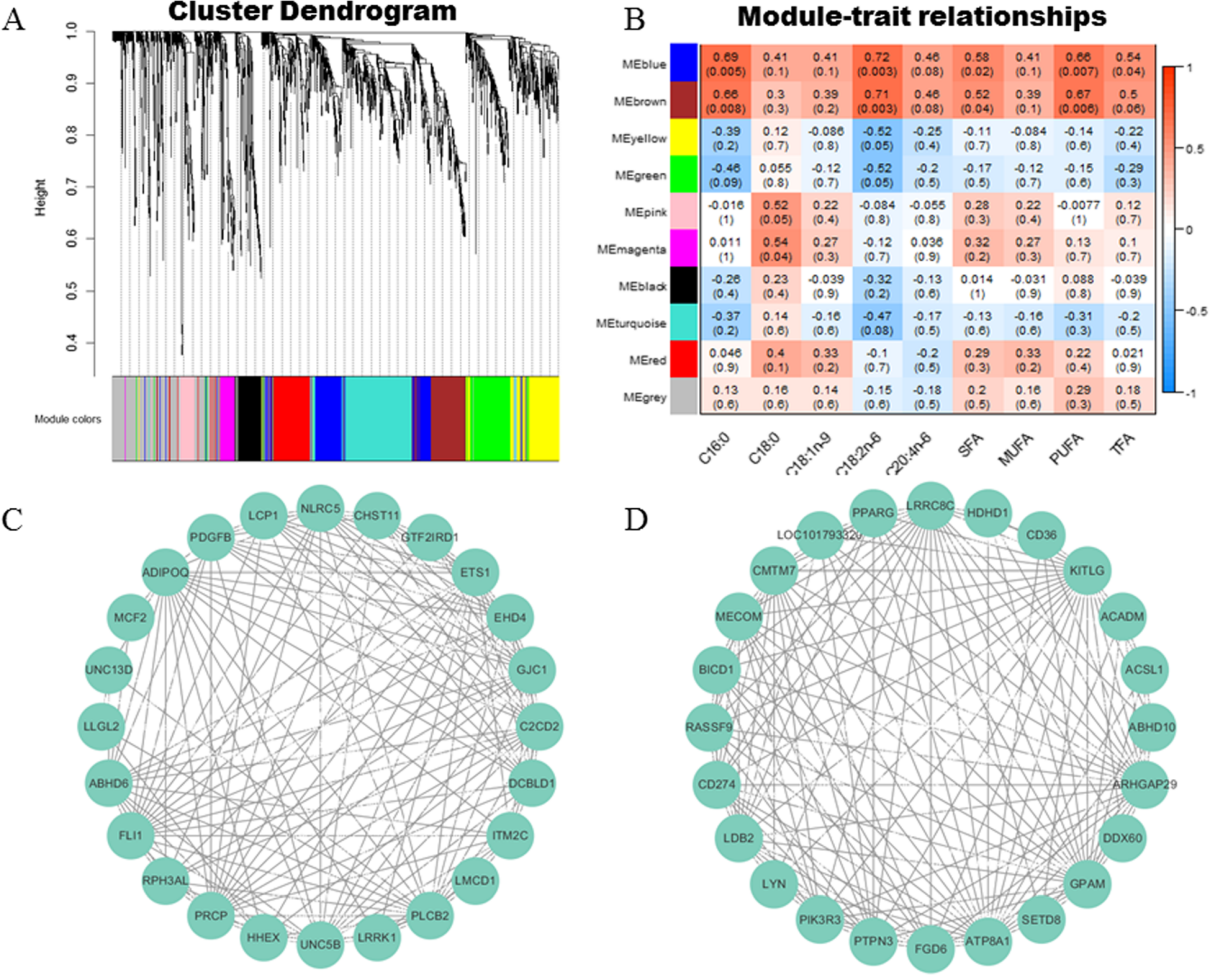
Detection of co-expression network in duck breast muscle**. a** Hierarchical cluster tree showing co-expression modules identified by WGCNA analysis. Each leaf in the tree is one gene. The major tree branches constitute nine modules labeled by different colors. **b** Module-tissue association. Each row corresponds to a module. Each column corresponds to a specific fatty acid composition trait. The color of each cell at the row-column intersection indicates the correlation coefficient between the module and the trait. A high degree of correlation between a specific module and the trait is indicated by dark red or dark green. **c** and **d** The relationships between the hub genes and lipid metabolism genes in MEblue and MEbrown. The top 150 connections sorted by correlation coefficients among transcripts are shown for each module

As a complementary approach to the single gene correlation analysis, we further investigated the correlation between network modules with the fatty acid composition traits. The 2024 DEGs were used for weighted gene co-expression network analysis (WGCNA) and nine co-expression modules were obtained (Fig. 5a). We calculated the correlation between module eigengene and nine fatty acid composition traits. Our result showed that the module MEblue and MEbrown significantly correlated with five fatty acid composition traits(C16:0, C18:2n-6, SFA, PUFA and TFA). MEpink and MEmagenta showed significant positive correlation with C18:0. While, MEyellow and MEgreen showed significant negative correlation with C18:2n-6 (Fig. 5b). We screened the genes in MEblue and MEbrown and found that a number of well-known lipid metabolism related genes such as peroxisome proliferator-activated receptor gamma coactivator 1-alpha (*PPARGC1A*), elongation of very long chain fatty acid 1 (*ELOVL1*), *CD36* and *ACADM* were included in these modules. We identified the hub genes in MEblue and and MEbrown for C16:0, and co-expression networks were constructed based on the expression coefficients of these hub genes and the lipid metabolism-related genes (Fig. 5c and d).

### Expression regulation of lipid metabolism related genes and its correlations with fatty acid composition traits

**Fig. 6:**
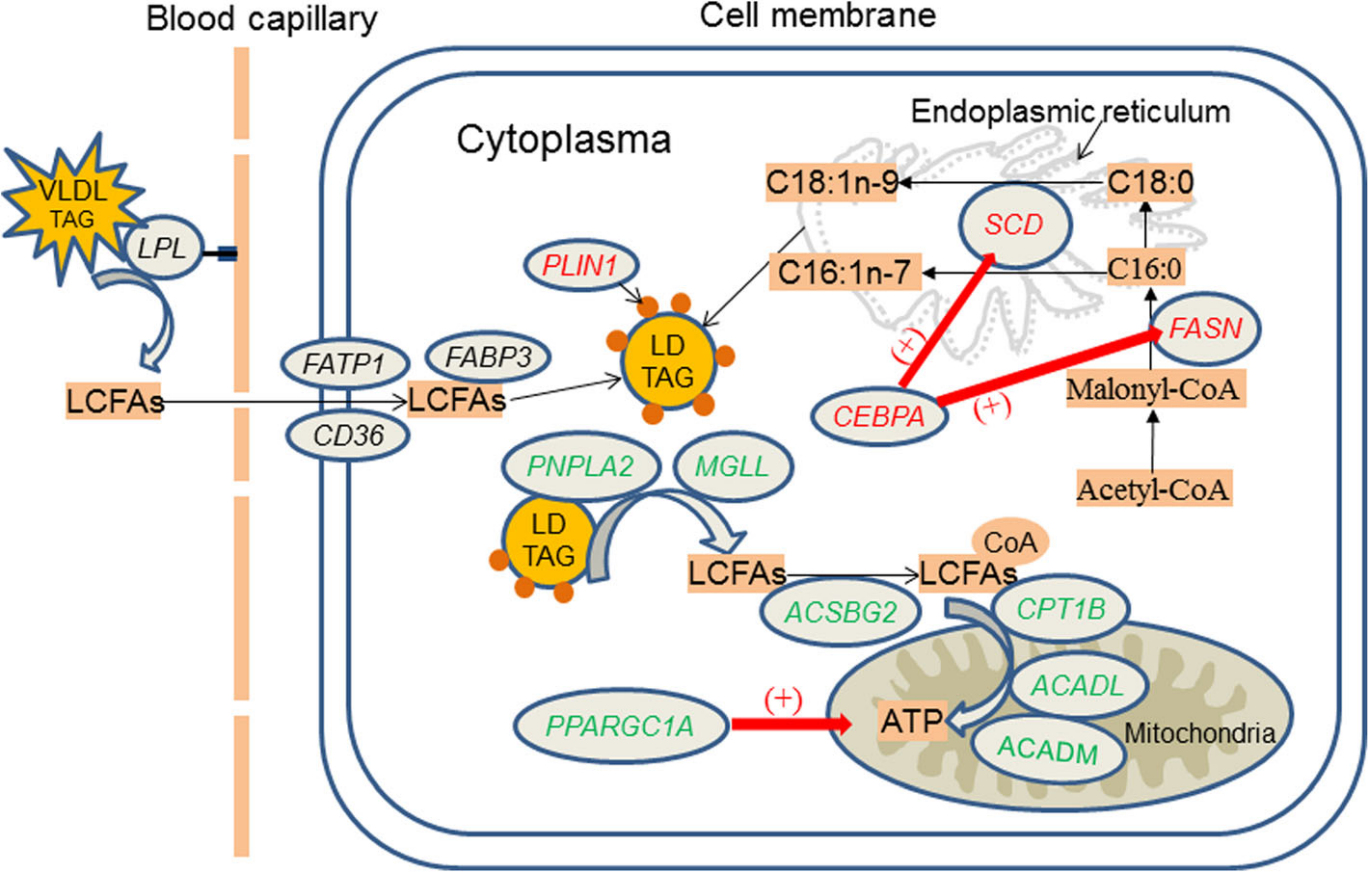
Summary of the differentially expressed genes involved directly or indirectly in lipid metabolism. Genes in green were highly expressed in mallards. Genes in red were highly expressed in Pekin ducks. Genes in black were not differentially expressed between the two breeds. The red arrows represent positive regulation by a transcriptor

**Fig. 7:**
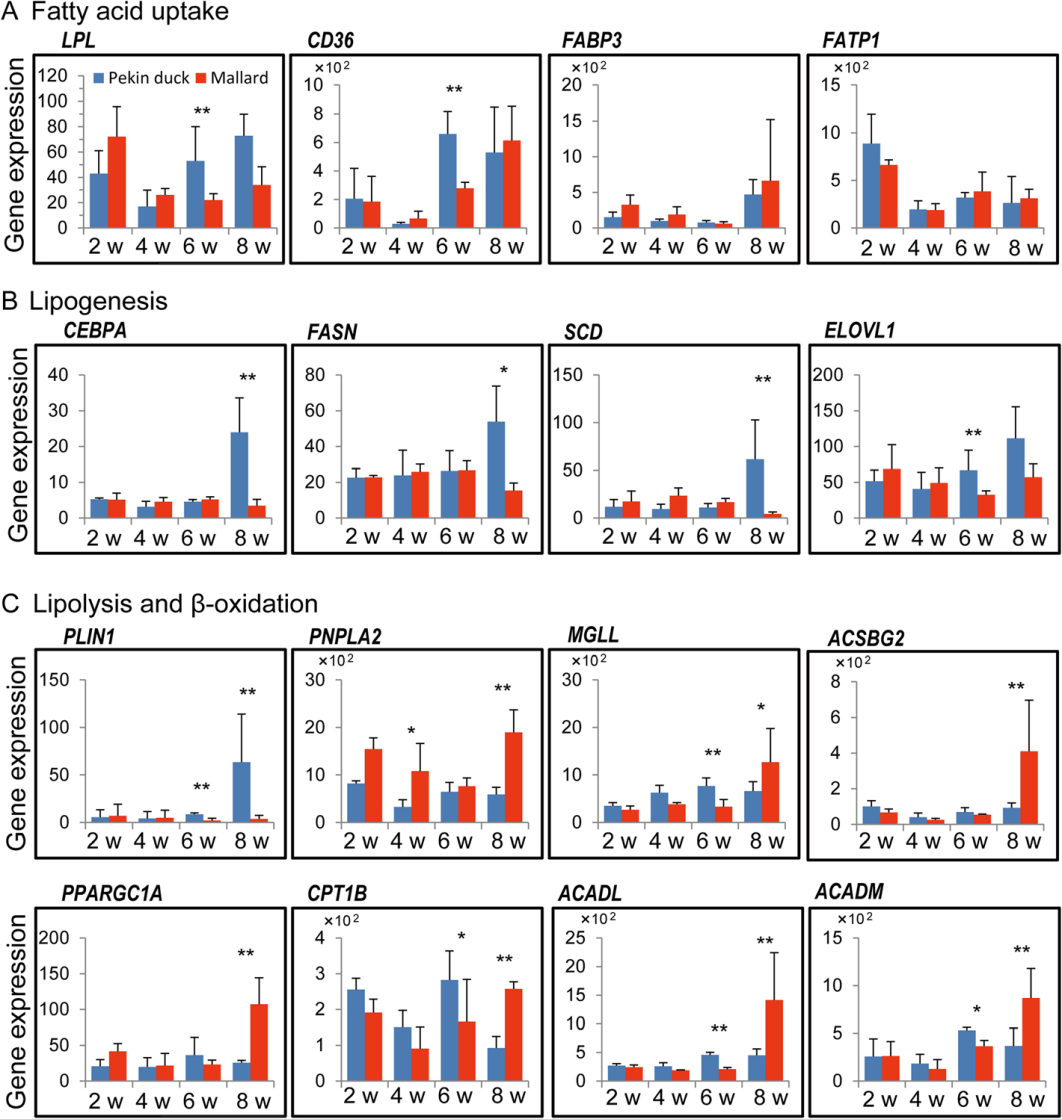
Expression regulation of genes involved in lipid metabolism. **a**–**c** Expression levels (CPM values) as determined from RNA-seq of genes involved in (**a**) fatty acid uptake, **b** lipogenesis, **c** Lipolysis and fatty acid *β*-oxidation. Expression levels are presented as averaged CPM at each time point (means ± SD, *n* = 3). * denote significance at *P* < 0.05 based on Fisher exact test. ** denote significance at P_adjust_ < 0.05 following Benjamin correction

**Fig. 8:**
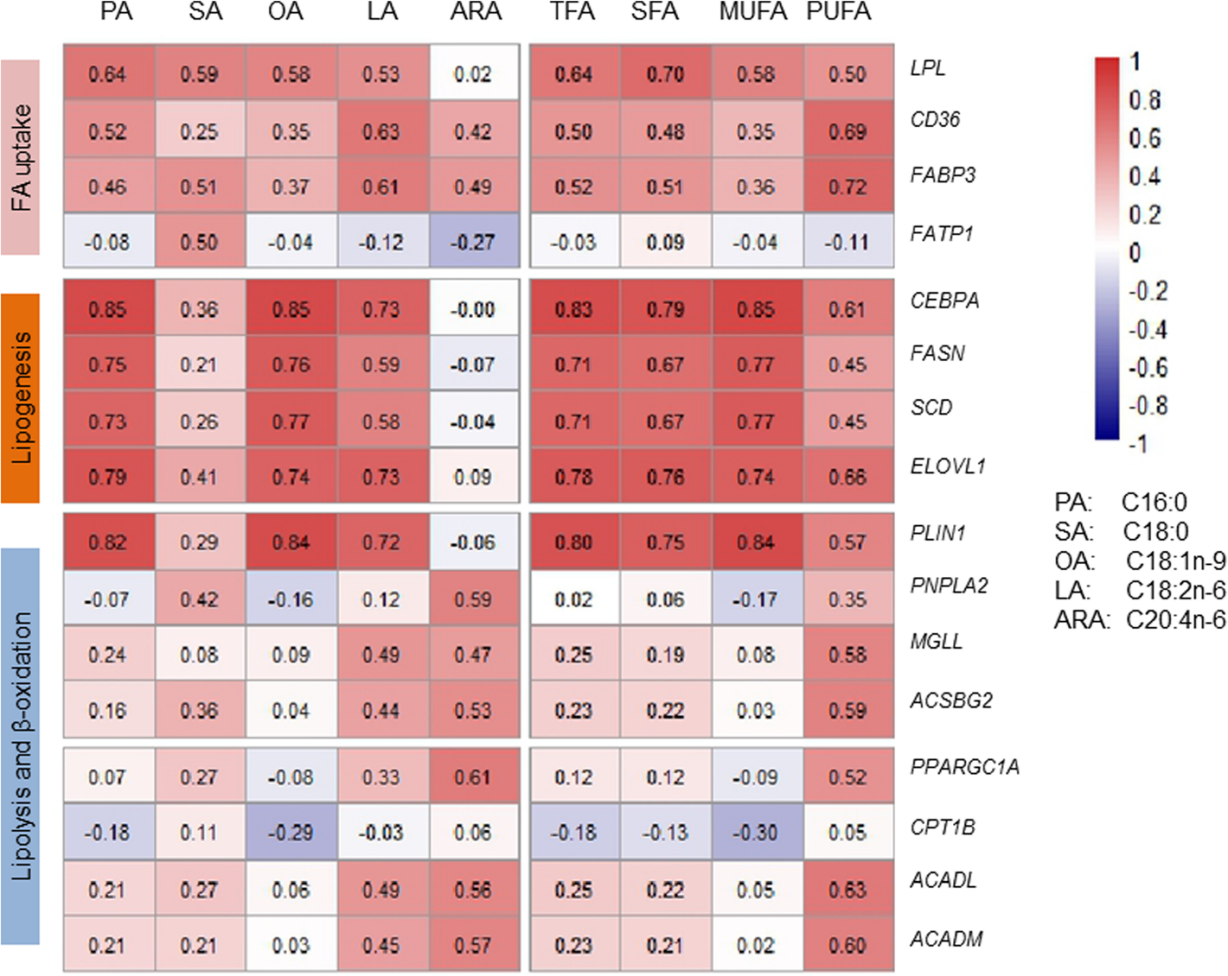
Correlations of selected genes with fatty acid composition traits. The number in each cell represents the correlation coefficient

The focus of the present study was on identifying the underlying mechanisms associated with differences in fatty acid accumulation between Pekin duck and mallard. A closer examination were conducted for expression regulation of genes involved in fatty acid uptake, lipogenesis, lipolysis and β-oxidation (Fig. 6 and 7). We found that expression regulation of these genes between Pekin duck and mallard mainly occurred at 6-weeks and 8-weeks. As shown in Fig. 7, the genes involved in lipogenesis were upregulated in Pekin duck at 8-weeks; whereas those involved in lipolysis and β-oxidation were upregulated in mallard at 8-weeks. The correlation between expression level of these gene and fatty acid composition traits was variable (Additional file 6). It was worth noting that the genes involved in lipogenesis showed strong positive correlation with C16:0, C18:1n-9 and C18:2n-6; whereas the genes involved in lipolysis and β-oxidation showed a strong positive correlation with C18:2n-6 and C20:4n-6(Fig. 8). Collectively, our results indicate that the regulation of fatty acid accumulation in duck breast muscle involves both lipogenesis and lipolysis.

## Discussion

Fatty acid composition contributes importantly to meat quality and is essential to the nutritional value of the meat. However, system-based understanding of fatty acid accumulation in poultry meat is lacking. For the present study, we reported for the first time the temporal progression of fatty acid accumulation in duck breast muscle and explored the correlations between fatty acid composition traits and global gene expression.

### Effect of age, sex and breeds on the accumulation of fatty acids in duck breast muscle

The deposition of fatty acids in meat was a complex and dynamic process, that could be affected by various factors such as age, sex, breed and rearing conditions of the animals. In the current study, we identified 20 fatty acids in duck breast muscle and found that the species and predominance order of indicated fatty acids were similar to previous reports [14, 27, 28]. We compared the composition of fatty acid between male and female ducks and found that it was really difficult to make a clear conclusion about the influence of duck sex on fatty acid composition of breast muscle. Previous reports about the influence of duck sex on the fatty acid composition of breast meat were also conflict. Some studies have demonstrated that duck sex has no influence on the fatty acid composition of breast meat [29, 30]. However, other study indicated that sex, as a main effect, had significant influence on proportions of C18:0, C18:1n-9, C18:2n-6, MUFA and PUFA [10]. Further studies were required to clarify the influence of duck sex on fatty composition of breast muscle in regard to ages and genotype.

Principal component analysis (PCA) of fatty acid concentration in this study revealed that both breeds and developmental stages have an influence on the deposition of fatty acids in duck breast muscle. In the present study, we observed that the contents of major fatty acids and fatty acid groups decreased dramatically from 2 weeks to 4 weeks, which was in agreement with a previous report on mule ducks [31]. We also found that the speed of fatty acid accumulation in duck breast muscle was the opposite of muscle fiber hypertrophy, which suggest that muscle fibers may obtain their energy requirements for growth and activity through lipolysis of their storage lipids, potentially explaining why lipid content decreased from 2 weeks to 4 weeks. Previous studies have demonstrated that the deposition of lipid in skeletal muscle was inversely related to body weight gain [31, 32].

It has been speculated in previous studies that the high levels of PUFAs (C20 and C22) in the meat of wild mallards mainly resulted from the birds’ diet, as these fatty acids can be derived from exogenous sources [14, 33]. In the present study, all the ducks were reared under the same conditions, suggesting that the different fatty acid profiles of the two breeds were mainly due to genetic variation between them. Indeed, the PUFAs are essential components of cell membranes, and the amount of which usually remains stable due to their important roles in membranes flexibility. While, the storage of energy through SFA and MUFA may change among individuals and over time. Therefor, the higher percentage of PUFAs and lower amount of storage fat (SFA and MUFA) in mallard may be the direct effect of a lower adipogenic potential.

### Expression regulation of genes involved in fatty acid uptake

Fatty acids derived from the blood circulation are one of the major sources of storage lipid in skeletal muscle. Fatty acid uptake in muscle is dependent on metabolic demands and lipid availability. Once inside the cell, fatty acids enter the oxidative process, or if fatty acid uptake exceeds fatty acid oxidation, they are used for triacylglycerol (TAG) synthesis, and stored in confined compartments, often lipid droplets (LDs) [34]. Several genes have been reported to be involved in fatty acid uptake in skeletal muscle, including *LPL*, fatty acid transport protein 1 (*FATP1*), *CD36* and *FABP3* [35, 36].

*LPL* is a key enzyme that hydrolyzes circulating triglycerides, and provides non-esterified fatty acids for tissue utilization [37]. *LPL* has been supposed to play an important role in regulating the uptake of fatty acid in a number of tissues, and up-regulation of *LPL* expression has been linked to increased muscle lipid uptake [38, 39]. *FATP1* and *CD36* are associated with the extracellular transport of fatty acids from capillary vessels into the cytoplasm, and *FABP3* is associated with the transport of fatty acid from cytoplasm to organelle membrane [40, 41]. However, our transcriptome analysis revealed no difference in the expression of these genes between the two breeds at all time points, except at 6 weeks where the transcript levels of *LPL* and *CD36* were higher in Pekin ducks than in mallards (Fig. 7a). We then screened for gene-trait correlations, and weak to moderate correlations were observed for *LPL*, *CD36* and *FABP3*, whereas *FATP1* showed very low correlations with all of the fatty acid composition traits, except C18:0 content, with which it showed a moderate correlation (Fig. 8). Therefore, it was difficult to build a correlation between the genes involved in fatty acid uptake and the higher content of fatty acid in breast muscle of Pekin duck than mallard.

### Expression regulation of genes involved in lipogenesis

In vitro studies have shown that lipogenesis plays a central role in lipid accumulation in muscle of mammals. De novo fatty acid synthesis (also referred to as de novo lipogenesis) occurs from the generation of C16:0 by the *FASN* in cytoplasm [42]. After the formation of palmitate, a series of chain elongations and desaturations occurs involving *SCD* and ELOVLs, to generate unsaturated or long-chain fatty acids [43, 44]. A previous study reported that *SCD* can have a strong effect on fatty acid composition within skeletal muscle by converting SFA into MUFA [20]. In the present study, the expression levels of both *FASN* and *SCD* were higher in Pekin ducks than mallards at 8 weeks, consistent with the finding that the Pekin ducks had higher contents of C16:0 and C18:1n-9 than did the mallard at 8 weeks. Of the *ELOVL*s detected in our data, *ELOVL1* was the most abundant and showed higher expression level in Pekin ducks than in mallards at 6 weeks and 8 weeks (Fig. 7b). In mammals, *ELOVL1* has been proposed to catalyze the formation of saturated and monounsaturated fatty acids containing 18–26 carbons [45]. In the present study, the expression of *FASN*, *SCD* and *ELOVL1* were strongly and positively correlated with C16:0, C18:1n-9 and C18:2n-6 contents, but weakly correlated with C18:0 and C20:4n-6 contents (Fig. 8).

The expression of genes involved in lipogenesis is tightly controlled by tissue-specific transcription factors [46]*. CEBPA* is among the well-known transcription factors involved in lipogenesis and adipogenesis and its activation is usually followed by increased fat deposition [47, 48]. In the present study, *CEBPA* showed higher expression level in Pekin ducks than in mallards at 8 weeks and strong correlations with the contents of C16:0, C18:1n-9 and C18:2n-6 (Fig. 7b, Fig. 8). These results suggest that the upregulated expression of *FASN*, *SCD* and *ELOVL1* may contribute to the high IMF content in Pekin ducks by increasing the synthesis of C16:0 and C18:1n-9 between 6 weeks and 8 weeks. The expression of these genes may be under the regulation of *CEBPA*.

### Expression regulation of genes involved in lipolysis and fatty acid β-oxidation

In the skeletal muscle of vertebrates, excess fatty acids are mostly stored as TAG in LDs. These LDs are coated with one or more of the perilipin family of proteins, which functions in stabilizing LDs and protecting them from lipolysis [34, 49]. *PLIN1* and *PLIN2* are the only two perilipin genes expressed in duck breast muscle, and *PLIN1* was expressed at higher levels in Pekin ducks than in mallards at 8 weeks (Fig. 7c). The levels of *PLIN*s have been shown to be correlated positively with LD content in the skeletal muscle of mammals [34]. Skeletal muscle is responsible for the body’s energy expenditure and fatty acids derived from the lipolysis of lipid droplets are the major fuel supply for muscle contraction. The genes patatin like phospholipase domain containing 2 (*PNPLA2*) and monoglyceride lipase (*MGLL*) encode the first and last enzymes involved in the hydrolysis of triglycerides and provide free fatty acids to tissues for *β*-oxidation. Overexpression of *PNPLA2* and *MGLL* is usually associated with increased oxidative capacity and decreased intramuscular lipid accumulation in skeletal muscle [50, 51]. In the present study, both genes were expressed at lower levels in Pekin ducks than in mallards at 8 weeks, indicating higher lipid lipolysis in the breast muscle of mallards than in that of Pekin ducks (Fig. 7c).

The degradation of fatty acids involves the activation of long-chain fatty acids, carnitine transport and fatty acid *β*-oxidation [36]. Several genes encoding rate-limiting enzymes were expressed at higher levels in mallards than in Pekin ducks at 8 weeks, including *ACSBG2*, carnitine palmitoyltransferase 1B (*CPT1B*), acyl-CoA dehydrogenase long chain (*ACADL*) and *ACADM* (Fig. 7c). *PPARGC1A* is a transcriptional coactivator that may regulate genes involved in mitochondrial oxidative metabolism and lower expression level of *PPARGC1A* in muscle has been reported in pigs with high content of oleic acid [21]. In the present study, *PPARGC1A* was expressed at higher levels in mallards than in Pekin ducks at 8 weeks, indicating that *PPARGC1A* may play a role in promoting fatty acid oxidation of duck breast muscle (Fig. 7c, Fig. 8).

The result of gene-trait correlations for genes related to lipolysis or β-oxidation yielded partially conflicting results. *PLIN1* showed strong correlation with C16:0, C18:1n-9 and C18:2n-6 contents, but weak correlations with C18:0 and C20:4n-6 contents. In contrast, the remaining genes showed weak correlations with C16:0, C18:0 and C18:1n-9 contents, but moderate correlations with C18:2n-6 and C20:4n-6 contents (Fig. 8). As mentioned above, although the causal relationship can not be obtained from gene–trait correlation analyses, we speculate that the differential expression of *PLIN1* may have occurred in response to the changes in SFA and MUFA. Whereas, the higher expression of other lipolysis-related or *β*-oxidation-related genes in mallards may have occurred in response to the increased level of PUFAs in this breed. This speculation is consistent with a previous report that pigs with higher PUFA levels tend to present higher expression of genes involved in lipolysis and fatty acid degradation, favoring the generation of ATP, mitochondrial function and oxidative capacity in muscles [52]. Therefore, it can be concluded that mallards have higher rates of lipolysis and fatty acid *β*-oxidation than do Pekin ducks, and that *PPARGC1A* may function in the regulation of genes involved in these processes.

## Conclusion

In summary, we reported the temporal progression of fatty acid accumulation and the dynamics of the transcriptome in breast muscle of Pekin ducks and mallards. Our results revealed that Pekin ducks have a stronger capacity than do mallards to accumulate SFAs (mainly C16:0) and MUFAs (mainly C16:1n-7 and C18:1n-9) between 6 weeks and 8 weeks. Correlation analysis of the abundance of DEGs and fatty acid composition traits revealed that *CEBPA* and *PPARGC1A* may function as regulators of lipogenesis, lipolysis and fatty acid *β*-oxidation and thereby influence the deposition of fatty acids in duck breast muscle. Our results provides insights into the transcriptomic regulation of fatty acid accumulation in duck breast muscle, and will facilitate the improvement of fatty acid composition in duck breeding.

## Materials and methods

### Animals and sample collection

A total of 150 mallard and 150 Pekin duck eggs were obtained from the experimental farm of Institute of Animal Sciences (CAAS, Beijing, China). All eggs were incubated using the normal procedure and all ducks were reared in cages under continuous lighting using standard conditions of temperature, humidity and ventilation at the farm of the IAS, CAAS. All ducks were fed the same corn- and soybean meal-based diet which met or exceeded the nutrient recommendations of National Research Council (NRC, 1994). Feed and water were provided ad libitum during the experiment (Additional file 7).

Animal handling and sampling protocols were in accordance with institutional guidelines. Following a 12-h overnight fast, 10 ducks (5 males and 5 females) of each breed were randomly selected and euthanized by CO_2_ asphyxiation and exsanguination at day of 14 (2 weeks), 28 (4 weeks), 42 (6 weeks) and 56 (8 weeks) after birth. The breast muscle from the left side was rapidly collected, immediately snap-frozen using liquid nitrogen and stored at − 80 °C. After this study, the remaining ducks were released to population for breed conservation.

### Histological evaluation

The pectoral muscles were fixed in 10% neutralized formalin and embedded in paraffin blocks. The muscle sections (5 μm) were stained with hematoxylin and eosin (H&E). For each bird, muscle fiber size was estimated by measuring the average diameter and area of at least 100 fibers using Image-Pro Plus 6.0 software (Media Cybernetics, Silver Spring, USA), and the density of muscle fibers (fibers/mm^2^) was estimated by point-counting stereology using 500 points.

### Measurement of fatty acid composition and oil concentration

Breast muscles were lyophilized and milled to a fine powder. The meat powder was analyzed for fatty acid composition using a gas chromatograph. Fatty acids was released from total lipids and methylated with methyl alcohol:acethyl chloride (10:1, v/v) according to a previous protocol [53]. The pentadecanoic acid was used as an internal standard. The 7890A GC-FID system (Agilent Technologies, Palo Alto, CA) equipped with a DB-23 column (Agilent Technologies, 60 m × 0.25 mm × 0.25 μm) was used to determine the FAME (Fatty acid methyl ester) profiles. Fatty acids were identified by comparison of their retention times with those of FAME standards (Supelco, 37 Component FAME mix C4-C24, Catalog No. 18919-1AMP, Supelco, Bellefonte, PA). All data were acquired on ChemStation software (Agilent Technologies) and normalized to sample weight and to the internal reference. Oil concentration was calculated as the sum of all identified fatty acid concentrations with percentage (%) of meat weight. In addition, fatty acids were indexed as groups of saturated, monounsaturated, polyunsaturated fatty acid, total of saturated fatty acid (SFA), total monounsaturated (MUFA), total of polyunsaturated (PUFA), total of omega 3 (n-3) and total of omega 6 (n-6). The calculation of various fatty acid groups are described as follows: SFA = C14:0 + C16:0 + C18:0 + C20:0 + C22:0 + C24:0; MUFA = C14:1n-5 + C16:1n-7 + C18:1n-9 + C20:1n-11+ C22:1n-13+ C24:1n-15; PUFA = C18:2n-6 + C18:3n-6 + C18:3n-3+ C20:2n-6 + C20:3n-6 + C20:4n-6 + C20:5n-3 + C22:6n-3; n-3 = C18:3n-3 + C20:5n-3 + C22:6n-3; n-6 = C18:2n-6 + C18:3n-6 + C20:2n-6+ C20:3n-6 + C20:4n-6; MUFA/SFA: ratio between MUFA and SFA; PUFA/SFA: ratio between PUFA and SFA; n-6/n-3: ratio between n-6 and n-3.

### RNA extraction, quality analysis, library preparation and sequencing

Total RNA was extracted from 50 mg of frozen breast muscle using TRIzol reagent (Takara, Dalian, China) following the manufacturer’s instructions for subsequent library preparation. The quantity and quality of total RNA was evaluated using the NanoDrop2000 system (Thermo Fisher Scientific, Illkirch, France) and evaluated for purity and integrity using the Bioanalyzer 2100 (Agilent Technologies, Santa Clara, CA, USA). All extractions used for sequencing yielded sufficient amounts of high-quality RNA for library construction. The mRNA was enriched from total RNA using oligo-(dT) magnetic beads and cDNA was synthesized by reverse transcription using a random hexamer-primer. Twenty-four libraries (3 Pekin ducks and 3 mallards for each time point) were produced for RNA-seq experiment and sequenced on an Illumina X ten machine using the 150-bp pair-end sequencing module. The average output was 6 Gb per library (Additional file 8).

Illumina RNA-Seq data for this study have been deposited at BIG Data Center (http://bigd.big.ac.cn/) with the accession codes PRJCA001307.

### Quality control and read alignment

Sequencing adaptors and low-complexity reads were removed in an initial data filtering step. Quality control and reads statistics were estimated with FASTQC version 0.10.1 software (http://www.bioinformatics.bbsrc.ac.uk/projects/fastqc/). The paired-end reads were mapped against the Pekin duck reference genome (http://www.duckbase.org/Download) using the Tophat version 2.0.11 software [54]. Subsequently, read counts per gene were obtained by running HTSeq version 0.6.1 software (http://www-huber.embl.de /users/anders/HTSeq/) [55]. CPM (counts per million mapped sequence reads) values were calculated for each gene model. Genes with averaged CPM among replicates ≥1 at no less than one time point were considered expressed and retained for further analysis.

### Identification of differential expressed genes and KEGG pathway analysis

Differentially expressed genes were identified using the edgeR statistical package available at Bioconductor open source software for bioinformatics. Before comparisons among samples of different library sizes and RNA composition, normalization was conducted using the “calcNormFactors()” function. Multiple corrections for the *P*-value were performed using the Benjamini-Hochberg’s approach for controlling the false discovery rate. Genes with a |log_2_(fold-change) | ≥1 and adjusted *p*-value < 0.05 were defined as differentially expressed genes (DEGs).

We formally characterized the functions of DEGs by searching for overrepresented pathways associated with these genes. First, we obtained the protein sequences of the DEGs. Then, we performed enrichment analysis of these genes using “Fasta Protein Sequence” by “Gene-list Enrichment” in KOBAS 3.0 [56]. “*Gallus gallus* (chicken)”, “hypergeometric test / Fisher’s exact test” and “Benjamini and Hochberg (1995)” were selected as “Species”, “Statistical method” and “FDR correction method” respectively.

### Quantitative real-time PCR (Q-PCR) analysis

To validate and characterize the DE transcripts identified by high-throughput sequencing, Q-PCR analyses were performed in an QuantStudio 7 Flex real-time PCR System (Life Technologie™). RNA samples were reverse transcribed to cDNA with the use of PrimerScript™ RT Master Mix (RR036A, Takara, Dalian, China) following the manufacturer’s instructions. The amplification was performed in triplicate in a total volume of 20 μl, containing 10 μl of 2 × TB Green Premix Ex Taq II(Tli RNaseH Plus, RR820A, Takara, Dalian, China), 1 μl of the diluted cDNA, and 0.5 μl of each primer, and 0.4 μl ROX Reference Dye II and 7.6 μl PCR-grade water. The real-time PCR program started with denaturing at 95 °C for 30s min, followed by 40 cycles of 95 °C for 5 s and 60 °C for 34 s. Data were analyzed with ABI Q7 software (V1.2) with the baseline being set automatically by the software. The relative mRNA expression level was calculated using the 2^−ΔΔCt^ method [57]. Results were expressed as the mean fold-change in gene expression, using the Pekin ducks at 6 weeks as the calibrator (assigned an expression level of 1). β-actin was used as the housekeeping gene and all primers of examined genes are described in Additional file 9.

### STEM clustering

Short Time-series Expression Miner software (STEM) was used to cluster and visualize possible profiles of DEG change in expression over time [58]. The maximum number of model profiles was adjusted to 40, and the maximum unit change in model profiles between time points was set to 1. Gene expression profiles were clustered according to correlation coefficient. The statistical significance of the number of genes assigned to each profile was computed by the algorithm described by Ernstet et al. [59].

### Correlation analysis of fatty acid composition traits and DEGs

Pearson correlation coefficients were calculated for the abundance of fatty acids or fatty acid groups and DEGs using pearsonr function in the Python package scipy.stats. A total of nine fatty acid composition traits (C16:0, C18:0, C18:1n-9, C18:2n-6, C20:4n-6, SFA, MUFA, PUFA and TFA) of 78 individuals and 2024 DEGs were subjected to Pearson correlation analysis. Samples with no expression data were assigned with the mean expression value of the corresponding developmental stage.

### Weighted gene co-expression network analysis

We applied Weighted Correlation Network Analysis (WGCNA) to construct gene modules with distinct expression patterns [60]. A total of 2024 DEGs were used for module constructions and nine co-expression modules were obtained. We assessed the relevance of co-expression modules with nine fatty acid composition traits using the Spearman’s correlation of the module eigengene with the trait. We defined an intramodular connectivity (Kin) measure for each gene based on its correlation with the rest of genes in a given module. The top 150 connections of each network was visualized using Cytoscape_3.7.1.

## Supplementary information

**Additional file 1 **Fatty acid profiles in breast muscle of Pekin ducks and mallards at ages of 2 weeks, 4 weeks, 6 weeks and 8 weeks**.**

**Additional file 2.** Difference of percentage and relative content of fatty acids between male and female ducks.

**Additional file 3 **Dynamics of fatty acids and fatty acid groups in breast muscle of Pekin ducks and mallards (means ± SD, *n* = 9 or 10). UFA/SFA represents the ratio of summed UFA with SFA. And, n-6/n-3 represents the ratio of summed n-6 with n-3 (values has no unit).

**Additional file 4.** Technical validation of RNA-seq results using Q-PCR by correlation analysis.

**Additional file 5.** List of Genes in significant profiles and KEGG pathway enrichment analysis for each profile.

**Additional file 6.** Correlations of fatty acid composition traits and DEGs.

**Additional file 7.** Composition and main calculated characteristics of diets.

**Additional file 8.** Summary of sample information used for transcriptomic analysis.

**Additional file 9.** The specific primers for Q-PCR in this study.

## Data Availability

The Illumina sequencing data used in this study can be available at BIG Data Center (http://bigd.big.ac.cn/) with the accession codes PRJCA001307. Other data sets supporting the results of this article are included within the article and its additional files.

## Abbreviations

DEGs: Differentially expressed genes
IMF: Intramuscular fat
KEGG: Kyoto Encyclopedia of Genes and Genomes
LDs: Lipid droplets
MUFA: Monounsaturated fatty Acid
PCA: Principal component analysis
PPAR: Peroxisome proliferator-activated receptor
PUFA: Polyunsaturated fatty Acid
SFA: Saturated fatty Acid
TFA: Total fatty acid
