# Supplementary figures and images for: Dynamic accumulation of fatty acids in duck (Anas platyrhynchos) breast muscle and its correlations with gene expression
Source: BMC Genomics. 2020 Jan 17;21:58. doi: 10.1186/s12864-020-6482-7 (PMC6969424; doi:10.1186/s12864-020-6482-7)

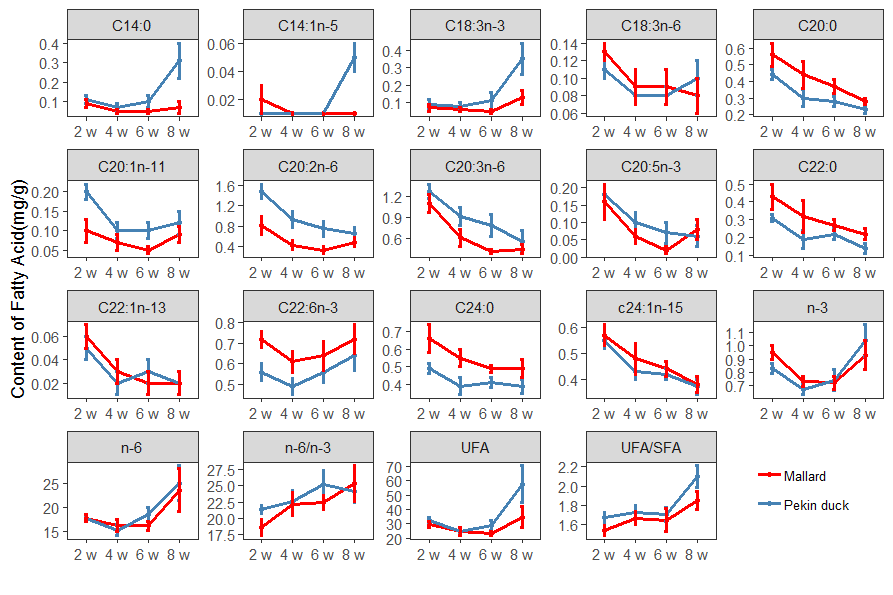

Supplement: Supplementary file 3 — Additional file 3 Dynamics of fatty acids and fatty acid groups in breast muscle of Pekin ducks and mallards (means ± SD, n = 9 or 10). UFA/SFA represents the ratio of summed UFA with SFA. And, n-6/n-3 represents the ratio of summed n-6 with n-3 (values has no unit). [file 12864_2020_6482_MOESM3_ESM.tiff]

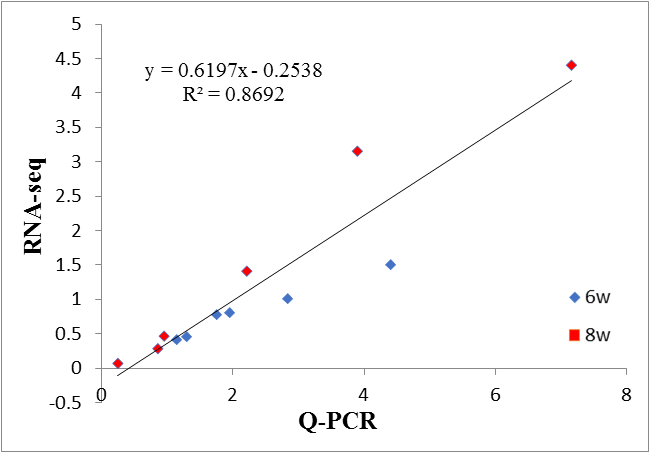

Supplement: Supplementary file 4 — Additional file 4. Technical validation of RNA-seq results using Q-PCR by correlation analysis. [file 12864_2020_6482_MOESM4_ESM.tif]
